# Polysaccharide sulfotransferases: the identification of putative sequences and respective functional characterisation

**DOI:** 10.1042/EBC20230094

**Published:** 2024-12-04

**Authors:** Ravina Mistry, Dominic P. Byrne, David Starns, Igor L. Barsukov, Edwin A. Yates, David G. Fernig

**Affiliations:** Department of Biochemistry, Cell and Systems Biology, Institute of Systems, Molecular and Integrative Biology, University of Liverpool, Liverpool L69 7ZB, U.K.

**Keywords:** algae, glycobiology, High-throughput assay, Pfam, sulfotransferases

## Abstract

The vast structural diversity of sulfated polysaccharides demands an equally diverse array of enzymes known as polysaccharide sulfotransferases (PSTs). PSTs are present across all kingdoms of life, including algae, fungi and archaea, and their sulfation pathways are relatively unexplored. Sulfated polysaccharides possess anti-inflammatory, anticoagulant and anti-cancer properties and have great therapeutic potential. Current identification of PSTs using Pfam has been predominantly focused on the identification of glycosaminoglycan (GAG) sulfotransferases because of their pivotal roles in cell communication, extracellular matrix formation and coagulation. As a result, our knowledge of non-GAG PSTs structure and function remains limited. The major sulfotransferase families, *Sulfotransfer_1* and *Sulfotransfer_2*, display broad homology and should enable the capture of a wide assortment of sulfotransferases but are limited in non-GAG PST sequence annotation. In addition, sequence annotation is further restricted by the paucity of biochemical analyses of PSTs. There are now high-throughput and robust assays for sulfotransferases such as colorimetric PAPS (3′-phosphoadenosine 5′-phosphosulfate) coupled assays, Europium-based fluorescent probes for ratiometric PAP (3′-phosphoadenosine-5′-phosphate) detection, and NMR methods for activity and product analysis. These techniques provide real-time and direct measurements to enhance the functional annotation and subsequent analysis of sulfated polysaccharides across the tree of life to improve putative PST identification and characterisation of function. Improved annotation and biochemical analysis of PST sequences will enhance the utility of PSTs across biomedical and biotechnological sectors.

## Introduction

Regioselective sulfation of polysaccharides is coordinated by a class of enzymes collectively referred to as polysaccharide sulfotransferases (PSTs). PSTs catalyse the sulfation (addition of SO_3_^−^) of primary or secondary hydroxyl (*O-*sulfation) or amino (*N*-sulfation) groups on polysaccharides and glycoconjugates, using the sulfate donor PAPS (3′-phosphoadenosine 5′-phosphosulfate). PSTs represent one of four subclasses of biological sulfotransferases (STs), which also include cytosolic sulfotransferases (SULTs) [1], PAPS-independent STs [1,2], and tyrosylprotein STs [3,4]. PSTs can be further delineated into glycosaminoglycan (GAG) and non-GAG STs. Owing to the emphasis on human health-related research, our understanding of sulfotransferase biology is heavily skewed towards GAG-directed enzymes and tyrosylprotein STs, in the areas of post-translational/biosynthesis modifications [5–8]. GAG STs, which target heparan sulfate (HS), chondroitin sulfate and keratan sulfate, have been studied in some depth, and are therefore not the primary focus for this review; interested readers are directed to recent reviews [3,9–11]. In contrast, there is comparably little known about putative non-GAG polysaccharide sulfotransferases.

Polysaccharides and glycoconjugates display great structural heterogeneity across all kingdoms of life. Homo- and heteropolysaccharide chains are composed of monosaccharide subunits, which can include and are not limited to, alternating combinations of d-glucose, l-fucose, d-galactose, l-galactose, d-mannose, l-arabinose, and d-xylose, amino sugars (d-glucosamine and d-galactosamine) and sugar acids (d-glucuronic, l-iduronic, and sialic acids) [12,13]. The structural complexity of polysaccharides is further enhanced by glycosidic linkage type, branched chains and secondary modifications (including sulfation, phosphorylation, methylation, and acetylation [13–15]). Of these, sulfation is a means of imparting a strongly acidic and negatively charged group to heteroatoms and induce pronounced change in the structural, physiochemical and biological properties of the polysaccharide [16]. Examples include HS and chondroitin sulfate (belonging to the GAG family), which regulate multiple aspects of cell communication in animals [3,10] and, in marine life, key constituents of algae cell walls are sulfated glycans [17–21]. Algal sulfated polysaccharides have also been shown to possess anti-inflammatory, anticancer and anticoagulant properties, and their ability to bind complementary surfaces of proteins akin to the action of GAGs, is a likely source of much of their biological activity [13,22–25]. In addition, they are employed across food, cosmetic and pharmaceutical products [25] due to their innate gelling properties [24]. When considering the enormous diversity of sulfated polysaccharides, it stands to reason that their synthesis is predicated on the emergence of a similarly vast array of carbohydrate active enzymes throughout evolutionary history. However, their structural heterogeneity has also complicated the identification of PSTs by their sequences alone.

**Figure 1:**
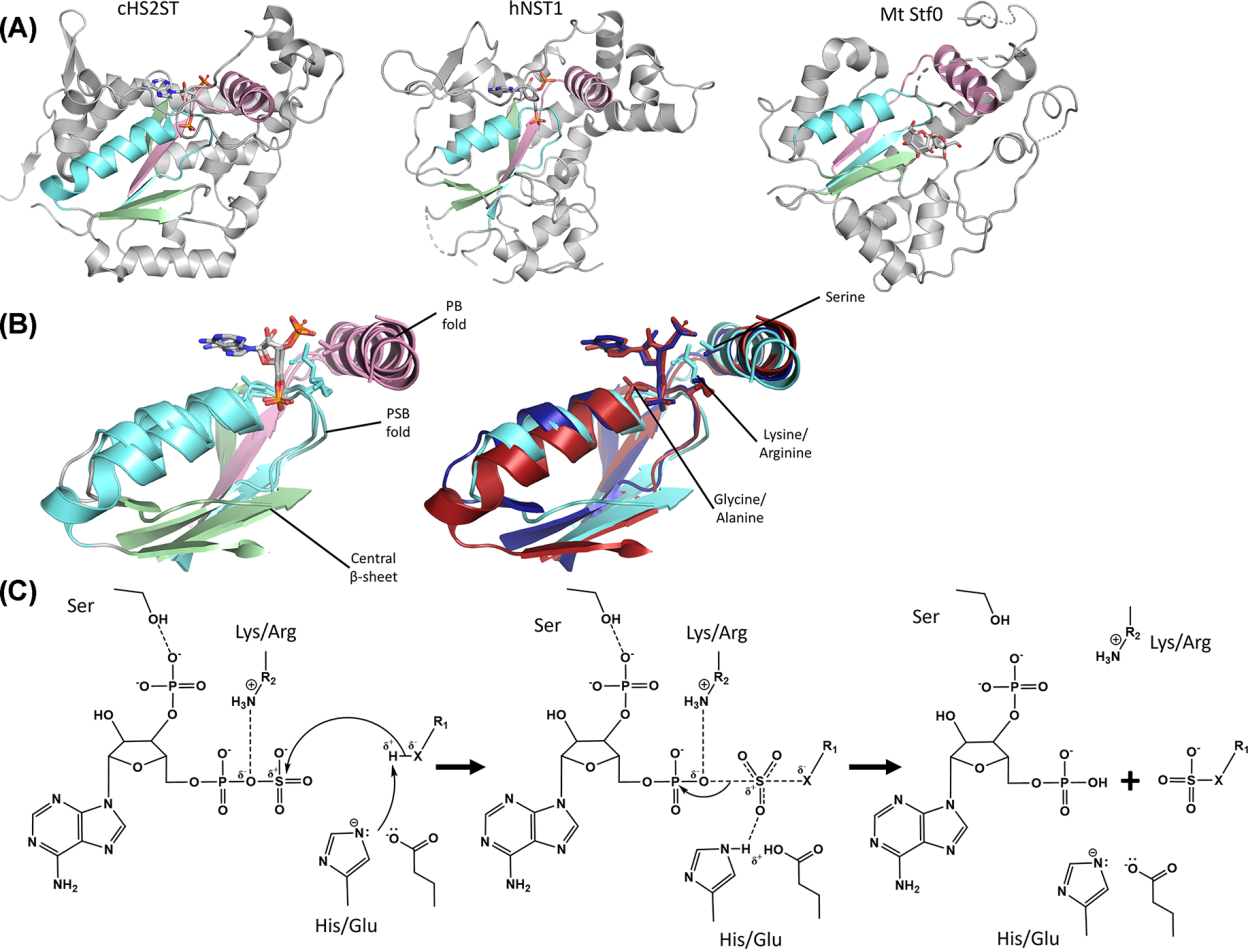
Overall sulfotransferase structure, PAPS binding site geometry comparison between cHS2ST, hNST and Mt Stf0, and proposed consensus mechanism of sulfate transfer (**A**) Similarity of the geometry of the PAPS-binding folds for cHS2ST, hNST1 and mycobacterium tuberculosis (Mt) Stf0 (PDB ID: 3F5F, 1NST and 1TEX [30,33,112]) with three conserved folds. The PAPS-binding fold, consisting of a strand-loop-helix containing the PAPS-binding loop (PSB, cyan), the strand-turn-helix for 3′ phosphate binding (PB, pink) and central β-sheet (green) is displayed. Large sections of the surrounding architecture are structurally distinct and display poor homology on sequence alignment. Key differences include the increased length of the PAPS-binding fold helix in cHS2ST and number of β-strands in each structure; cHS2ST and hNST1 have five strands in their central β-sheet, whereas Mt Stf0 only displays three strands. This may be attributed to the truncated genome of mycobacterium tuberculosis. (**B**) The geometry of the core PAPS-binding folds, coloured as in (A), is highly similar on removal of the surrounding architecture, and annotated with fold name and with the position of conserved residues between Pfam families PF00685 and PF03567 on the right. These folds typically contain glycine and lysine on the PSB and a conserved serine on the α helix of the PB fold; with the exception of Mt Stf0 which contains alanine and arginine on the PSB (cHS2ST, red; hNST1, dark blue; Mt Stf0, cyan). Structures were superimposed in Pymol 2.1.1. ( **C**) Consensus mechanism of PST sulfate transfer via an SN2-like mechanism. Concerted attack of the H bound to a heteroatom from the catalytic base (histidine or glutamate) directs the heteroatom to attack S within sulfate. This results in a trigonal bi-pyramidal transition state with increased coordination of the S. The ether bond is stabilised by lysine, resulting in the O remaining with the phosphate, with sulfate transfer occurring in a single displacement reaction. R1: monosaccharide; R2: lysine or arginine.

PSTs have drawn interest as potential green catalyst for the production of custom sulfated oligosaccharide, but such an approach first requires identification of novel PSTs with specific function to maximise their catalytic exploitation [26–28]. Although the structures of several PSTs have been solved (Figure 1A) [29–35], determining PST structures has often proven challenging due to difficulties in producing sufficient yields of proteins for crystallographic investigations [10]. Recent advances in predictive computational biology, such as AlphaFold2 [36], have empowered our functional understanding of PSTs and are amenable to synthetic biology strategies to support the generation of enzymes with desirable biochemical properties (such as genetically stabilised protein variants [10,37]). These advances have positioned PSTs as viable targets for use across biotechnology and biomedical industries. This review highlights sequence identification and methodologies that can assist in the functional characterisation of PSTs, and collates investigation of sulfated products in lesser-studied organisms to expand the known utility and perceived significance of PSTs.

### Polysaccharide sulfotransferase structure and Pfam identification

**Figure 2:**
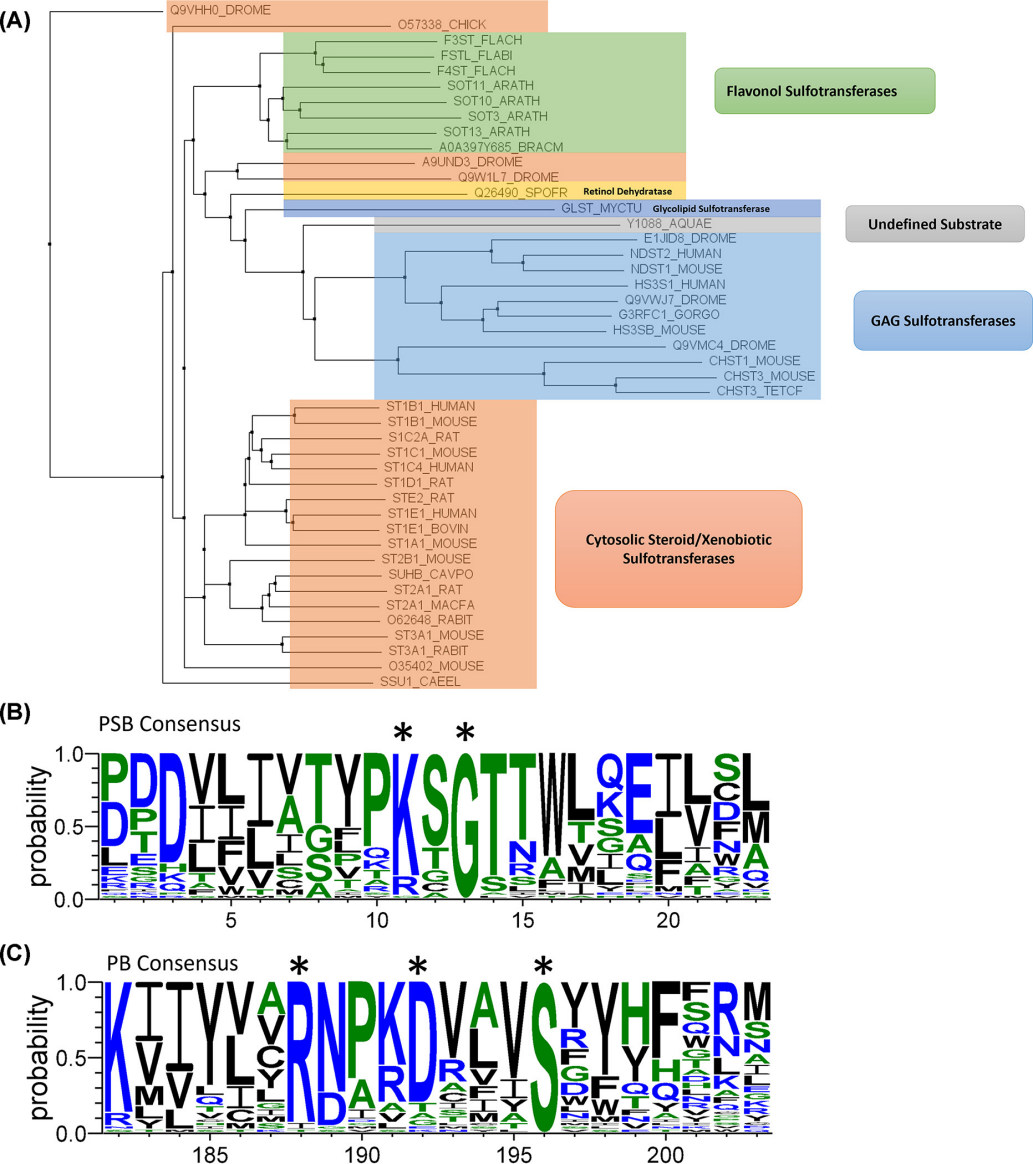
Functional annotation and consensus sequences for the PAPS binding loop (PSB) and 3' phosphate binding site (PB) of *Sulfotransfer_1* (PF00685) seed (**A**) PF00685 seed annotated with the function of sequences currently used to identify putative sulfotransferases. Annotated sequences are predominantly cytosolic aryl sulfotransferases (orange), GAG sulfotransferases (blue) and plant flavonol sulfotransferases (green). A unique retinol dehydratase is also included, which utilises PAPS to remove a hydroxyl from retinol (yellow) [113]. The PF00685 seed contains 45 sequences, identified by their Uniprot ID, and are displayed as a phylogenetic tree constructed using the neighbour joining method in Jalview 2.22.3.2. (**B**) The consensus sequence of positions 1-23 of the PF00685 seed alignment, representing the strand-loop-helix of the PAPS-binding fold, containing a highly conserved lysine and glycine, marked with an asterix. (**C**) The consensus sequence of positions 182-203 of the PF00685 seed alignment, representing the strand-turn-helix for 3′ phosphate binding, producing a RXXXDXXXS consensus motif, marked with an asterix. Panels (B,C) were created using WebLogo 3.7.12, with amino acid colour relating to hydrophobicity (Neutral, green; Hydrophilic, blue; Hydrophobic, black) [114].

**Figure 3:**
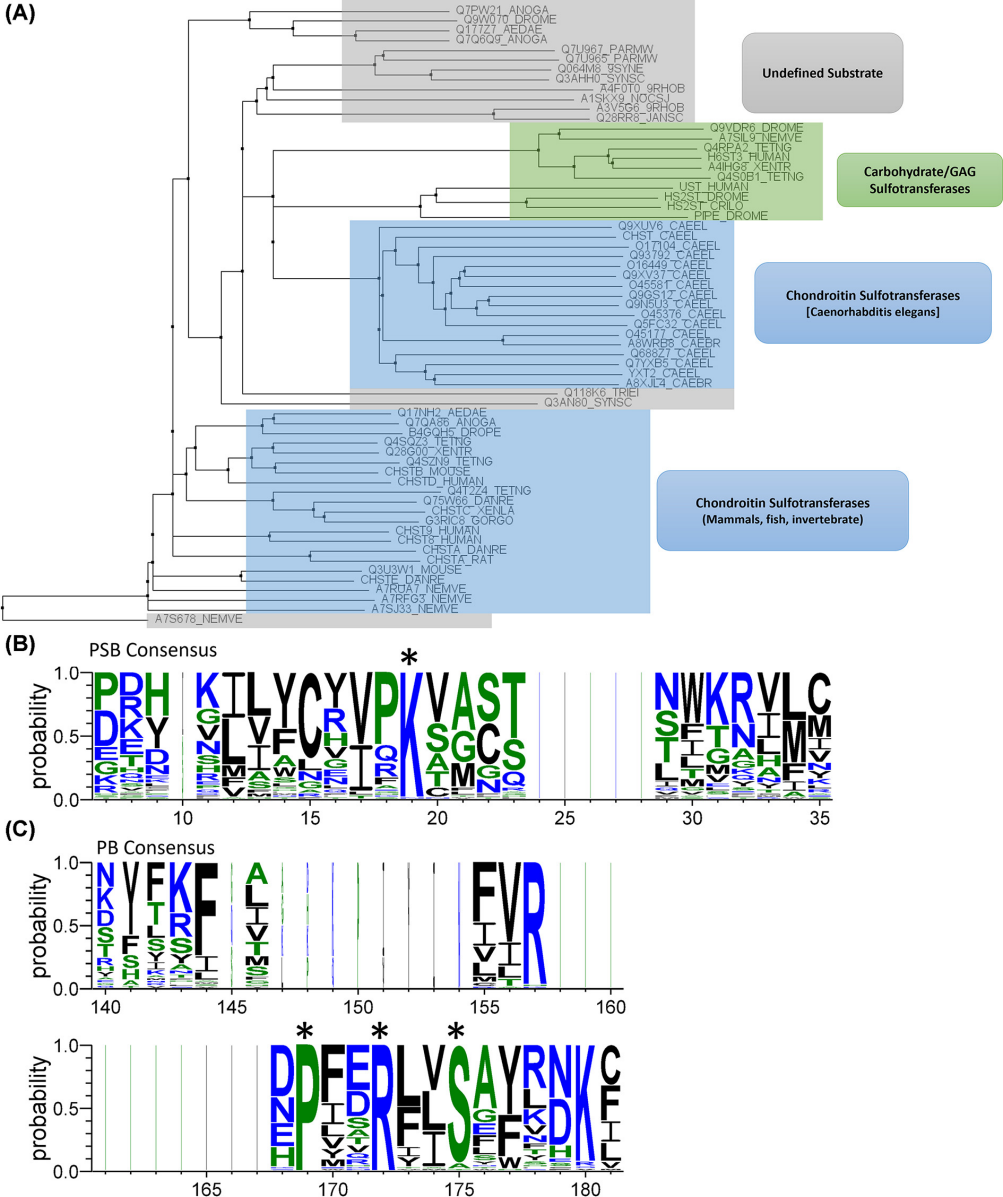
Functional annotation and consensus sequences for the PAPS binding loop (PSB) and 3' phosphate binding site (PB) of *sulfotransfer_2* (PF03567) seed (**A**) PF03567 seed annotated with the function of sequences currently used to identify putative polysaccharide sulfotransferases. Annotated sequences are predominantly chondroitin sulfate sulfotransferases (blue), heparan sulfate sulfotransferases (green) and undefined sulfotransferases (grey). PF03567 seed contains 63 sequences, identified by their Uniprot ID, and are displayed as a phylogenetic tree constructed using the neighbour joining method in Jalview 2.22.3.2. (**B**) The consensus sequence of positions 7-35 of the PF03567 seed alignment, representing the strand-loop-helix of the PAPS-binding fold, containing a highly conserved lysine, which is marked with an asterix. (**C**) The consensus sequence of positions 140-181 of the PF03567 seed alignment, representing the strand-turn-helix for 3′ phosphate binding, producing a PXXRXXS consensus motif, marked with an asterix. Panels (B,C) were created using WebLogo 3.7.12, with amino acid colour relating to hydrophobicity (neutral, green; hydrophilic, blue; hydrophobic, black) [114].

Several structural features are common to PST activity. PST structure adopts a β-sheet flanked by α helixes (Figure 1A) [3], where this central β-sheet, comprised of three to five β-strands, is a key architectural feature designed to orientate the strand-loop-helix (containing the PAPS-binding loop [PSB]) and strand-turn-helix (containing the 3′ phosphate binding [PB] site) (Figure 1B). The folds superimpose well following the removal of non-conserved regions (Figure 1B) and act to position PAPS in an appropriate configuration for catalysis. The PSB contains a highly conserved catalytic lysine (Figures 2B and 3B), or arginine (in the case of mycobacterial glycolipid sulfotransferase [Mt Stf0]), which interacts with the ester between the 5′ phosphate and sulfate group to stabilise the transition state (Figure 1C) [3]. The second is a serine residue adjacent to the catalytic pocket, located on the PB helix (Figure 1B). Finally, a conserved histidine, or glutamate positioned on a non-conserved loop (in the case of hHS3ST1 and hNST1 [*N*-sulfotransferase domain of *N*-deactylase/*N*-sulfotransferase 1]), serves as a catalytic base to deprotonate an acceptor substrate [3,38]. Comparison of HS STs catalytic base and PAPS-binding folds highlights similarity between NSTs, HS3STs, and TPSTs compared with HS6STs, HS2STs, and SULTs [3]. In addition, biochemical analysis of several HS STs correlates conserved residues (Figure 1B) to enzymatic sulfate transfer and are consistent with catalysis occurring through a SN2-like mechanism via a trigonal bipyramidal transition state (Figure 1C) [3,31,32,38–41]. In some cases, water may play a significant role during sulfate transfer by stabilising this transition state, inferred from radial distribution function studies of hNST1, that identified two solvation sites within the active site [40].

**Figure 4:**
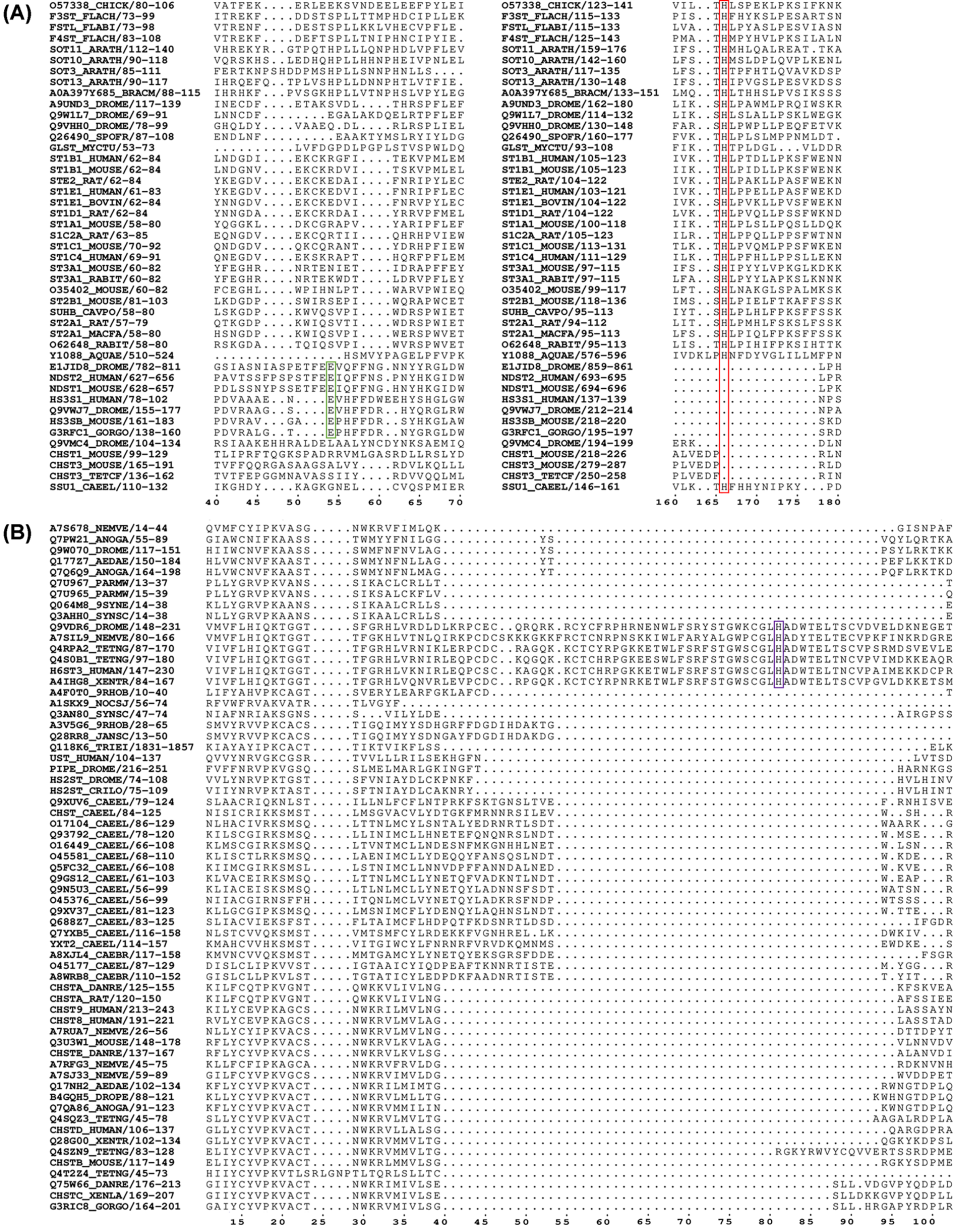
Position of catalytic base for HSSTs within *Sulfotransfer_1* (PF00685) and *Sulfotransfer_2* seed alignments (**A**) PF00685 seed demonstrating that catalytic base glutamate (green box), for HS STs, are not aligned with the conserved catalytic base histidine (red box). (**B**) PF03567 seed demonstrating that the catalytic base histidine is within non-conserved regions (purple box) relative to all sulfotransferase sequences retrieved by the seed. The ruler below the alignment notes the position of the amino acid in relation to all the sequences in the seed, and sequences are identified by their Uniprot ID. Seeds were accessed from Interpro from their respective entry (Table 1) and visualised in ESPript 3.0 [115].

This characteristic and evolutionary conserved PAPS-binding fold (Figure 1B) is the hallmark of PSTs and can be used to identify novel sulfotransferases based on sequence homology, using curated databases such as Pfam (hosted on Interpro). Pfam uses seed alignments to produce a scoring-profile employing Hidden Markov Models to infer functions for distantly related sequences [42]. PSTs can be identified with *Sulfotransfer_1* domain (PF00685) and *Sulfotransfer_2* domain (PF03567) which are the most reported families across the literature for identifying novel PSTs (Table 1) [22,43–46]. While sequence-based approaches have been instrumental in identifying mammalian GAG sulfotransferases, their effectiveness with non-mammalian organisms is constrained by current Pfam profiles. This stems from three factors: (I) the low sequence diversity of the seed may produce an unintended preference for GAG sulfotransferases; for example, 11 GAG sulfotransferases and 38 chondroitin sulfotransferases are present within PF00685 and PF03567 seeds, respectively (Figures 2A and 3A). This bias in detection potentially limits the identification of PSTs in bacteria, algae and fungi that do not display consensus PSB motifs (Figures 2B and 3B) or PB motif (Figures 2C and 3C) [47]. (II) The existence of large regions of non-conserved structure and sequence that are dedicated to substrate recognition (Figures 1A and 4B) and (III) inclusion of sequences with no functional annotation within seeds, further hinder homology-based identification of sulfotransferases (Figures 2A and 3A).

**Table 1:** Sulfotransferase domains and families entries hosted on Interpro, with profiles from Pfam [42], Panther [59] and Protein Information Resource [60]

Accession	Name	Type	Integrated Signatures	GO Terms
IPR000863	Sulfotransferase (Sulfotransfer_1)	Domain	PF00685	GO:0008146
IPR005331	Sulfotransferase (Sulfotransfer_2)	Family	PF03567	GO:0008146, GO:0016020
IPR007669	Carbohydrate sulfotransferase Chst-1	Family	PTHR22900	GO:0047756, GO:0030206, GO:1902884
IPR007734	Heparan sulphate 2-*O*-sulfotransferase	Family	PTHR12129	GO:0008146, GO:0016020
IPR009729	Galactose-3-*O*-sulfotransferase	Family	PF06990, PTHR14647	GO:0001733, GO:0009247, GO:0016020
IPR010635	Heparan sulphate 6-sulfotransferase/Protein-tyrosine sulfotransferase	Family	PTHR12812	GO:0008146, GO:0016020
IPR015124	Trehalose 2-sulfotransferase	Family	PIRSF021497	GO:0016740
IPR016469	Carbohydrate sulfotransferase	Family	PIRSF005883	GO:0008146, GO:0005975, GO:0000139
IPR018011	Carbohydrate sulfotransferase 8-10	Family	PTHR12137	GO:0008146, GO:0016051, GO:0016020
IPR024628	Sulphotransferase Stf0 (Sulfotransfer_0)	Domain	PF09037	
IPR025710	L-cysteine S-thiosulfotransferase subunit SoxA	Family	PIRSF038455, TIGR04484	GO:0009055, GO:0016669, GO:0020037, GO:0019417, GO:0070069
IPR026634	Protein-tyrosine sulfotransferase-like	Family	PTHR12788	GO:0008476, GO:0006478
IPR037359	Heparan sulfate sulfotransferase	Family	PTHR10605	GO:0008146
	Sulfotransferase domain (Sulfotransfer_5)	Domain	PF19798	
	Sulfotransferase family (Sulfotransfer_3)	Domain	PF13469	
IPR040632	Sulfotransferase, S. mansonii-type (Sulfotransfer_4)	Family	PF17784	
IPR010262 IPR039535 IPR028610	Arylsulfotransferase (ASST) Arylsulfate sulfotransferase AssT, Enterobacteria	Repeat	PF05935, PF14269, MF_00933	GO:0004062

The mechanism of sulfate transfer may be another potential differentiator between subclasses of sulfotransferases. Currently, the catalytic mechanism is not considered in ST classification as neither PF00685 nor PF03567 prioritise the position of the catalytic base in their seed alignments (Figure 4), thereby restricting the sulfotransferase definition to the conserved PSB lysine/arginine (Figures 2B and 3B), PB helix serine (Figures 2C and 3C) and β-sheet architecture of the PAPS-binding site.

### Polysaccharide recognition and identification of novel PST by substrate specificity

**Figure 5:**
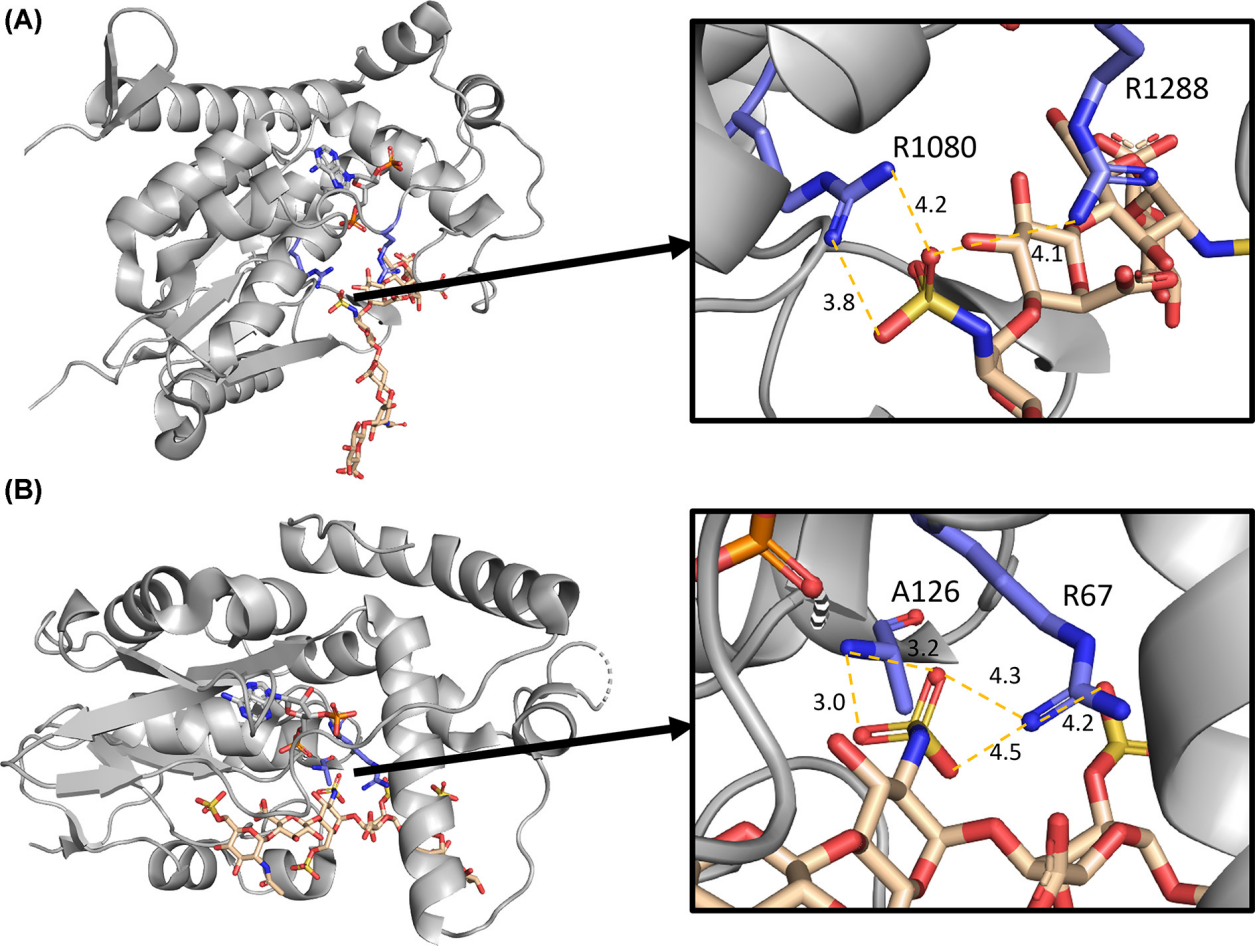
*N*-sulfation is a positional prerequisite for substrates of cHS2ST1 and HS2ST1 (**A**) cHS2ST1 (PDB ID 4NDZ) and (**B**) hHS3ST1 (3UAN) [34,35]). *N*-sulfate binding occurs though an active site arginine (distance from the sulfate is represented as Å). The length and flexibility of the arginine/lysine sidechain, the position of which can vary in an 8 Å radius, is of note. Residue numbers correspond to the position of the residue in the crystal structure.

A distinguishing feature of PSTs is their ability to perform regioselective sulfation, which requires tailored substrate recognition surfaces that vary between PSTs that sulfate virgin polysaccharide and those that modify pre-sulfated polysaccharide chains. This specificity is observed for cHS2ST, hHS3ST1, and mHS6ST3, which only recognise GlcNS containing substrate (Figure 5) [26,29] and, as seen with cHS2ST1, reject 6-*O* sulfation of glucosamine [34]. Typically, sulfotransferases interface with their cognate substrates through a combination of hydrogen bonding, electrostatic interaction and salt bridges [48,49], and STs that act in the final steps of GAG biosynthetic pathways, such as hHS6STs, sulfate on a GAG with greater hydrophilicity, where the polyelectrolyte effect plays an integral role in substrate binding and dissociation [49–51]. Specific residues involved in substrate recognition are determined through mutational analysis [29,30,35,52], as well as molecular dynamics simulation [40,48,49,53]. However, the structure–function relationships that drive substrate recognition of most PSTs remains ambiguous, reducing the predictive power of *in silico* analysis. Furthermore, it is important to consider that simulations require informed preparation of the system for a specific substrate, due to the high conformational and chemical flexibility of oligosaccharides [40,48,49,53]. Binding kinetics studied in tyrosylprotein STs and hHS6ST3 required the binding of PAPS to occur prior to substrate binding, displaying ordered sequential binding kinetics [3]. However, acceptor-substrate and PAPS binding can also be independent of the other, exemplified by Mt Stf0, which exhibits a random sequential Bi-Bi kinetic profile in the presence of PAPS and trehalose [54]. This kinetic mechanism has proven advantages from an evolutionary perspective, as it enables accumulation of adaptive mutation within the PAPS or substrate binding site without compromising the function of the other site, and this is evinced by the expanded repertoire of carbohydrate substrates in enzymes that utilise PAPS. Moreover, this plasticity can allow for the rational design of polysaccharide and aryl sulfotransferase PAPS-binding sites to improve catalysis, utilise alternative sulfate donors, such as APS (adenosine-5′-phosphosulfate) and *para*-nitrophenol sulfate (*p*NPS), whilst retaining acceptor substrate specificity, as demonstrated in [55–58].

Pfam classifications of sulfotransferases have developed considerably in recent years, due in part to a ‘relaxation’ of the pfam families that previously restricted overlap [42], and the massive increase in genome sequencing across the tree of life. Interpro now holds 17 entries for sulfotransferases, consisting of 8 pfam families and 11 additional families grouped by substrate specificity, in addition to PAPS binding (Table 1) [42,59,60]. However, the paucity of data on acceptor substrates means the latter families are limited to a few well-characterised polysaccharide sulfotransferases. Accommodation of a diverse range of carbohydrate substrates has necessitated the evolution of enzymes with highly variable conformational architecture, and as such sulfotransferases with different substrate specificities display poor sequence homology (Figure 4B). Improved genome annotation methodologies [22,44,61,62] and machine learning/neural network models can overcome some of the inherent limitations of pfam. For example, algorithms utilising the CAZy repository, a database of carbohydrate active enzymes, enabled the characterisation of substrate by protein fold, which has been applied to the annotation of glycosyltransferases and sulfatases [63,64]. A similar approach is likely to be profitable in the identification and classification of carbohydrate sulfotransferases.

## Assays: enzymatic and biochemical characterisation of PSTs

### Real-time PAP measurement assays

**Figure 6:**
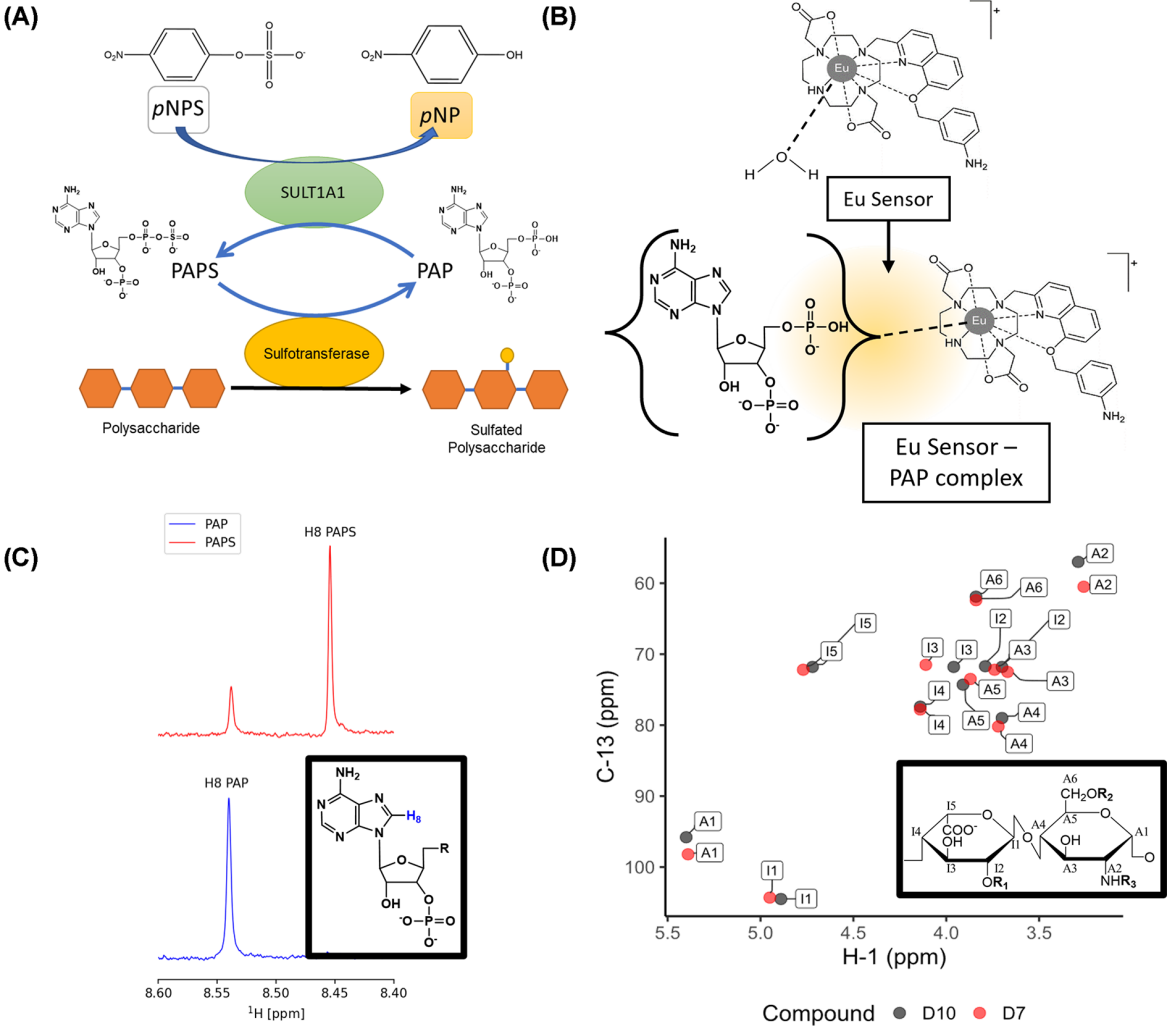
Biochemical assays for real-time measurement of PAP production (PAPS coupled assay, europium (III) sensor and ^1^H NMR) and the use of 2D NMR for product analysis (**A**) Schematic of PAPS coupled assay. PAP, produced as a consequence of the PST action, is regenerated by SULT1A1 to PAPS by transfer of the sulphonyl group of *p*NPS, generating *p*NP, which has a strong absorbance at 405 nm. (**B**) The europium (Eu) complex is a europium (III) ion chelated by a 8-(benzyloxy)quinoline functionalised with a meta-amino group on the benzene ring. There is a vacant coordination site on the Eu (III) ion, bound to water, which is displaced on binding with PAP causing luminescence [72]. (**C**) ^1^H NMR of H8 used to measure PAPS hydrolysis. The region of the spectra shown is between 8.6 ppm to 8.4 ppm to display the H8 protons of the adenine ring. The resonance of H8 is affected by the sulfonyl group and is at 8.46 ppm for PAPS and 8.55 ppm for PAP [72]. PAPS/PAP backbone is shown to indicate the position of proton H8, responsible for signals seen in ^1^H NMR. The R group of PAP would be PO_4_^2−^ and PO_4_^−^SO_3_^−^ in PAPS. (**D**) Chemical shift values collected [77] and visualised to display the pattern of change when the HSQC spectra are overlaid. Fully desulfated heparin (D10) peaks are in black and 2O, 6O desulfated heparin (D7) in red. Peaks are labelled by compound and assigned carbon from A1-A6 and I1-I5 in accordance with the disaccharide of predominant repeating structure of heparin derivatives. Nomenclature is according to Yates *et al*., (1996) [77], where A denotes glucosamine, and I denotes iduronic acid. The numerals represent the carbon atom within each monosaccharide residue. R_1_: -H, -SO_3_; R_2_: -H, -SO_3_; R_3_: -H, -SO_3_, -Ac.

Accurate biochemical characterisation of putative sulfotransferases also requires robust and high-throughput analytical techniques to expedite substrate classification. ST activity was formerly assessed using radiolabelled PAP^35^S, however this technique is discontinuous [65]. Enzyme assays that measure PAPS turnover are now available, applicable to all sulfotransferase classes. For instance, continuous ST-coupled assays utilise a PAPS regenerating enzyme (such as SULT1A1 [a mammalian cytosolic sulfotransferase]) which catalyses the transfer of a sulfate group from *p*NPS to PAP (3′-phosphoadenosine-5′-phosphate), resulting in accumulation of a *p*NP (*para*-nitrophenol) product which is monitored as a rising absorbance at 405 nm (Figure 6A) [66,67]. Concomitant PAP generation due to carbohydrate substrate sulfation by a secondary sulfotransferase can therefore be followed indirectly in this simple colorimetric assay. Continuous assays of this nature, where PAPS depletion is not a limiting factor to ST activity, can also amplify sulfated product yields, which can then be validated by 2D NMR [68]. Potential limitations of such an indirect method include non-productive PAPS conversion (including spontaneous PAPS hydrolysis and sulfate transfer to an off-target substrate [69]); greater *p*NP absorbance intensity at high pH [70], *p*NP toxicity [10,55] and the sulfation of assay components (enzyme, substrate, SULT1A1) by SULT1A1.

Although amenable to a high throughput format, coupled assays are less appropriate when screening for small molecule competitive inhibitors of a particular sulfotransferase. A recent adaptation of a microfluidic enzyme assay technology, that uses fluorescently labelled oligosaccharide acceptors elegantly circumvents this problem by directly monitoring substrate sulfation in real-time [71]. However, as this assay specifically detects changes in the electrophoretic mobility of a structurally defined oligosaccharide, that may be costly or difficult to synthesise, its utility for substrate discovery is limited. Luminescent europium (Eu) III probes provide the solution to these problems. Direct binding of the probe to PAP increases its luminescent intensity proportionally with the rate of PAPS consumption (Figure 6B) [72,73], obviating the requirement for secondary PAPS regenerating enzymes or sulfate donating compounds. However, as with coupled assays, the Eu(III) probe only detects PAP, where confirmation of direct substrate sulfation in either assay format would need to be independently verified using alternative analytical strategies (such as NMR, HPLC, gel-based methods, and thin layer chromatography) [23,74].

### NMR analysis of sulfotransferase activities

^1^H NMR can differentiate between PAP and PAPS, by monitoring chemical shifts at the H8 position of PAPS/PAP which produce signals at 8.46 and 8.55 ppm, respectively (Figure 6C) [72]. PAPS conversion by an ST is therefore detected as a loss of 8.46 ppm signal and a reciprocal gain at 8.55 ppm [72]. Alternatively, fluorinated PAPS analogues have also been used to monitor real-time sulfotransferase activity using ^19^FNMR [75]. However, again, this is an indirect assessment of activity and secondary verification of sulfation is required.

Fortunately, 2D NMR provides sufficient resolution to detect direct modifications of polysaccharides and has advantages over mass spectrometry (problems arise from the labile nature of the sulfate moiety during ionisation) and time-consuming HPLC analysis (which requires the use of high-grade, NMR defined standards) [23,71,74,76–78]. The spectra derived from native and sulfated substrates can be contrasted in order to assign the position of a sulfate group (Figure 6D); however, the accuracy of this is dependent on a number of factors relating to the degree of polymerisation, sulfation stoichiometry, and physiochemical properties of the polysaccharide under different buffer conditions [77,78]. For example, incomplete or partial sulfation leads to substrate heterogeneity, and consequently result in convoluted NMR spectra that are challenging to decipher. Of note, sulfation can drive changes in polysaccharide tertiary structure and impact enzyme binding, also leading to an incomplete sulfation profile.

## Polysaccharide sulfotransferases across the tree of life

The presence of specific polysaccharide classes and the spatial distribution of sulfate modification in living organisms can be a powerful prognostic tool to identify genetically encoded STs. The inference of unidentified ST(s) can be formed as a mechanistic explanation for the existence of unique sulfated polysaccharides, which could not exist without appropriate catalytic processing. By surveying the literature for uncommon sulfated polysaccharides, it is possible to predict the function of STs within poorly characterised genomes.

### Polysaccharide sulfotransferases in eukaryotes

Within vertebrates and invertebrates, the production of GAGs is well-documented [79] owing to their integral roles in extracellular matrix function, coagulation, growth, and development [15,80–82]. A recent study has also demonstrated the importance of sulfated sugars for the initiation of sexual reproduction, where the extracellular digestion of chondroitin sulfate induces swarming of choanoflagellates, revealing an ancient and evolutionary significant function in the last common unicellular ancestor of metazoans [43]. Although invertebrate GAGs serve similar functions to those found in vertebrates, evolutionary variation of GAG sulfation patterns or chain regularity has occurred to support differential clotting regulation, attributed to the fundamental difference in their internal or external environments [83,84]. Interestingly, marine organisms of high salinity habitats tend towards higher sulfation patterns compared to their terrestrial counterparts, which may indicate a dependency on a compensatory amplified charge density within high-electrolyte extracellular matrices [85]. Another organism that displays a rare sulfation trait is the giant African snail, which produces acharan sulfate with IdoA2S in the absence of GlcNS, a configuration not typically found in sulfated GAGs of mammals [86–89]. This offers a potential route to uncover more IdoA sulfotransferases.

Detection of GAG STs in other eukaryotes may eventually be achieved through the addition of sequences of lesser-studied organisms, for example, archaea and fungi. At present, classification of fungal ST genes is challenging due to their exclusion from current Pfam definitions and limited studies of their sulfated polysaccharides; with the exception of lichen, that have been found to produce sulfated polysaccharides for water retention [23]. However, with the use of organism specific databases, Interpro entries (IPR000863, IPR010262, IPR026634, IPR037359, IPR039535, IPR040632 [59]) found 1,747 sulfotransferase genes in FungiDB [90], predominantly in Aspergillus species, which also included putative HS sulfotransferases (IPR039535, Table 1).

### Non-eukaryotic STs and unusual sugar modifications

PSTs are required by all forms of life, with sulfation and phosphorylation occupying subtly different physiochemical niches and exhibiting alternative regulatory functions; the former mostly associated with extracellular signalling and serving to improve solubility of its biological conjugates and sequester environmental toxins or metal cations; and the latter predominantly associated with allosteric regulation of intracellular protein signalling cascades and the structures of nucleic acid polymers [16]. Sulfated glycans have also been discovered in archaea, including a rare IdoA(3S) containing N-linked glycan in *Halobacterium salinarum* and several sulfated N-linked glycans that decorate integral membrane proteins of *Thermoplasma acidophilum*, found to also contain an unusual direct C-S linkage (6-C-sulfo-d-fucose) [91,92]. Sulfation of IdoA occurs almost exclusively at the C2 position in GAGs, which may indicate that IdoA(3S) modification is only associated with archaea [91]. However, the STs responsible for this rare modification within the archaea family detected by PF03567 (such as *Euryarchaeota archaeon*, *Candidatus Pacearchaeota* archaeon, and *Candidatus Woesearchaeota* archaeon) have yet to be characterised and demonstrated to possess sulfotransferase activity. Fascinatingly, the envelopes of giant viruses of the *Megavirinae* subfamily contain many complex glycans and branched polysaccharides, and genes predicted to encode putative sulfotransferases have also been discovered [92]. Although further functional characterisation is required, it is tempting to speculate that sulfate modification of glycans may play a role in viral–host interactions.

#### Glycolipid sulfotransferases

Glycolipid STs have been heavily studied in vertebrates and have an important role in cerebroside glycolipid synthesis. These sulfotransferases are found using PF06990 (Gal-3OST); although this seed has derived a profile from just three sequences, it may still prove a great resource to identify PST sequences for lipophilic substrates if expanded further [93]. In this regard, another glycolipid sulfotransferase NodH (present in *Sinorhizobium meliloti* and *Rhizobium meliloti*) that is required to synthesise sulfated glycolipids and stimulate nodule formation of its symbiotic host, was initially identified using traditional biochemical techniques [94,95]. NodH lacked consensus with PF00685 and PF03567 profiles and is identified with PF13469 (Sulfotransfer_3, Table 1) due to an RXG motif in the PSB [94,95]. NodH possesses GlcNAc(6S) activity that is independent of *N*-sulfation, in stark contrast with typical HS biosynthesis [94]. In addition to this, six genera of bacteria are now reported to synthesise sulfated polysaccharides, and a *Mesorhizobium loti* glycolipid sulfotransferase, KpsS, was revealed to possess fucosyl ST activity [47]. As mentioned previously, the conserved mycobacterial glycolipid sulfotransferase Stf0, present in PF00685, also displays unique C2 trehalose ST activity, and plays a critical role in the first step of sulfated glycolipid SL-1, which is involved in the immunomodulatory pathology of mycobacterium tuberculosis infections [33,96]. Glycolipid STs are found across several Pfam families, where it may be prudent to acknowledge glycolipid sulfation as a distinct modification in itself within the Pfam repository, as bioinformatic sequence analysis indicates that glycolipid sulfation may occur across a further 12 genera of bacteria [47].

### Algae and macroalgae: home of carrageenan, fucoidan, and ulvan sulfotransferases

Microalgae and macroalgae are classified into Rhodophyceae (red), Phaeophyceae (brown), and Chlorophyta (green) and produce a diverse range of sulfated polysaccharides, such as carrageenans, fucoidans, and ulvans, respectively [24,25,44,97–99]. Sulfated polysaccharides are highly abundant in algae, with essential functions within the extracellular matrix and water retention properties to defend against desiccation stress [22,23,25]. The breadth of photosynthetic organisms that produce novel sulfated polysaccharides is extensively covered in Lee and Ho (2022) [13]. Mining of 9 algal genomes for PSTs, across the three lineages, saw the identification of 83 genes using both PF00685 and PF03567 [46] (Table 1). This demonstrated an unlikely ST speciation profile, where Chlorophyta PSTs were detected by PF00685, Rhodophyceae PSTs were predominantly detected with PF03567 and PF06990, and Phaeophyceae PSTs detected by all three Pfam families [46] (Table 1). This separation may be due to the three distinct sulfated polysaccharides predominantly produced by each phylum.

One of the major bioproducts of the green macroalgae, Chlorophyta, is ulvan, a branched sulfated polyanionic heteropolysaccharide [97,99]. The genera *Ulva* and *Enteromorpha* possess ulvans which are predominantly composed of rhamnose, xylose, and glucuronic acid and decorated with C3 sulfation on rhamnose and C2 sulfation on xylose [97]; whereas *Codium* contain sulfated arabinose polysaccharides (C2 and C4) and sulfated arabinogalactans (C4 and C6) [99,100]. Seemingly, this would necessitate this lineage of algae to possess either rhamno-3OSTs and xylo-2OSTs, or arabinose and galactose 4OSTs and 6OSTs, but neither have been identified.

The genomes and transcriptomes of three Phaeophyceae, *Saccharina japonica, E. siliculosus,* and *Cladosiphon okamuranus*, have been characterised with 44, 41, and 24 sulfotransferase genes found, respectively [62,101,102]. Given that fucoidans, or sulfated fucans, are predominantly found within Phaeophyceae, this may suggest that some of these sulfotransferases possess fucosyl ST activities. Fucoidans display 1-2, 1-3, and 1-4 linked L-fucose, with varied sulfation at positions C2, C3, and C4 [25,103,104]. The inherent heterogeneity of fucans may therefore require the concerted activities of several STs with divergent substrate specificities.

Carrageenans and agars (sulfated galactans) are predominantly found in Rhodophyceae macroalgae [105,106]. Carrageenans consist of a linear assembly of α(1-3), β(1-4) d-galactose, with sulfate modifications located on the C2, C4, and C6 positions of galactose [107]. The genome of Rhodophyceae *Chondrus crispus* contains several genes predicted to be involved in the metabolism of sulfur, including twelve candidate carrageenan sulfotransferases and homologous genes for sulfo-lipid synthesis [44]. This would strongly implicate the involvement of galactose directed STs in carrageenan synthesis, supported by putative PST sequences detected with both PF03567 and PF06990 in Rhodophyceae [46,106].

### Terrestrial plants

The relative paucity of biochemical studies and crystal structures constrains PST identification in plants. For a long time, polysaccharide sulfation was considered to be predominantly an algal trait due to its function in water retention, and to be less prevalent in terrestrial plants which adapted to reduced environmental salinity and relative sulfate scarcity [22,108,109]. Contrary to this, current evidence suggests that polysaccharide sulfation may be more common in terrestrial plants than originally anticipated, with sulfated polysaccharides documented and characterised in the roots of freshwater plants and species such as *Pimpinella anisum* seeds, stem lettuce and *Artemisia tripartite* [13,25,45,110]. Of note, the flowering species *Globularia alypum* produces a polysaccharide comprised of galactose, glucose, and mannose with 13% sulfate content [13,111]. However, genome sequencing is still required to identify the sulfotransferases involved in this biosynthetic pathway.

## Conclusion

Our current understanding of PSTs is seen through the lens of cytosolic and GAG sulfotransferases found in mammals. Broadening the functional characterisation of sulfotransferases to include algae and fungi, in addition to expanding the Pfam families to categorise sulfotransferases by substrate specificity would enhance the current generation of sequence tools to identify novel sulfotransferases. Routes to overcome the limitations of Pfam may see the classification of PSTs integrated with a CAZy database, similar to the classification of sulfatases and glycosyltransferases [63,64]. Analysis of sulfated polysaccharide production may also encourage predictions of sulfotransferase function, in tandem with the utilisation of high-throughput sulfotransferase assays to facilitate greater sequence annotation of PSTs. These methods have wide applicability to enhance drug discovery processes and support the bioengineering of PSTs for biotechnology.

## Summary

- Structural features common to sulfotransferases include folds for PAPS-binding loop and 3′ phosphate binding, positioned with a central β-sheet, however crystal structure information of non-GAG PSTs is limited.
- *Sulfotransfer_1* may identify PST due to the inclusion of GAG STs in this definition; however, *Sulfotransfer_1* is predominantly defined by cytosolic sulfotransferases that conjugate aryl substrates and hydroxyl groups.
- *Sulfotransfer_2* is defined predominantly by putative chondroitin sulfotransferases, thereby introducing detection bias towards mammalian-like GAG STs, and at present cannot ascertain non-GAG carbohydrate substrates nor the position of sulfation.
- Novel real-time and high-throughput assays, such as colourimetric coupled assays, fluorescent probes and NMR techniques enable the functional characterisation of novel putative polysaccharide sulfotransferases.

## Open Access

Open access for this article was enabled by the participation of University of Liverpool in an all-inclusive *Read & Publish* agreement with Portland Press and the Biochemical Society under a transformative agreement with JISC.

## Abbreviations

APS: adenosine-5′-phosphosulfate
GAG: glycosaminoglycan
HS: heparan sulfate
PAP: 3'-phosphoadenosine-5′-phosphate
PAPS: 3’-phosphoadenosine 5’-phosphosulfate
PB: 3′ phosphate binding site
*p*NPS: *para*-nitrophenol sulfate
PST: polysaccharide sulfotransferase
PSB: PAPS binding loop
ST: sulfotransferase
SULT: cytosolic sulfotransferase
